# A hidden side of the COVID-19 pandemic in children: the double burden of undernutrition and overnutrition

**DOI:** 10.1186/s12939-021-01390-w

**Published:** 2021-01-22

**Authors:** Boutaina Zemrani, Mario Gehri, Eric Masserey, Cyril Knob, Rachel Pellaton

**Affiliations:** 1 General Pediatric Unit, Department Woman-Mother-Child, Lausanne University Hospital, Lausanne, Switzerland; 2 Deputy Cantonal Doctor, Public Health Department, Canton of Vaud, Lausanne, Switzerland; 3 Pediatric Unit, Hôpital Riviera-Chablais, Rennaz, Switzerland

**Keywords:** COVID-19, Children, Impact, Inequality, Undernutrition, Obesity

## Abstract

The COVID-19 pandemic has deteriorated key determinants of health and caused major upheavals around the world. Children, although less directly affected by the virus, are paying a heavy price through the indirect effects of the crisis, including poor diet, mental health impact, social isolation, addiction to screens and lack of schooling and health care, particularly among vulnerable groups. This paper is aimed at discussing the potential impact of this pandemic on children’s nutrition and lifestyle. Preliminary data from the literature and from our survey show significant disruptions in nutrition and lifestyle habits of children. While undernutrition is expected to worsen in poor countries, obesity rates could increase in middle- and high-income countries especially among precarious groups widening the gap in health and social inequalities.

The real impact of the COVID-19 pandemic on children extends well beyond that of a viral infection. This crisis has public health implications that could have life-long consequences on children. It requires effective and targeted measures mainly for vulnerable children and households to guarantee children’s basic rights for optimal nutrition, health and development.

## Background

In 2019–2020, the world has witnessed the emergence of a new Coronavirus Disease, called COVID-19, which has turned the globe upside down and disrupted main determinants of health. In children, the most serious risks posed by the COVID-19 crisis are not those of the disease itself, but its collateral damages. These include inadequate nutrition with a risk of both overweight and underweight, addiction to screens, lack of schooling, mental health impact, social isolation, risk of child abuse and declining vaccination rates and health care [1].

The COVID-19 pandemic has shifted dietary and lifestyle habits of many families. In addition to the lockdown, the major economic turmoil has disrupted livelihood activities for millions of people worldwide, particularly those working in the informal economy. High-income countries, including the wealthiest, have witnessed shocking scenes of thousands of desperate persons queuing for long hours, week after week, to receive free food parcels; most are vulnerable persons issued from migration and low-income households. School closures also led to the interruption of programs allowing children from poor families to receive free or subsidized school lunches and healthy snacks exposing millions of children to food insecurity [2, 3]. In low-income countries, disruptions in nutrition assistance programs and health services add to deep-rooted poverty [4–6]. Financial struggles related to COVID-19 may push more families into poverty and may further force families to ration food and make cheaper and unhealthy food choices in order to pay for other needs such as rent and medications.

It is currently unknown to what extent this pandemic will impact the nutritional status of children around the globe, but it could compromise diet quality, quantity and diversity increasing the risk of various forms of malnutrition, namely obesity, undernutrition and hidden hunger due to micronutrient deficiencies, especially among vulnerable groups [3]. This commentary aims to shed the light and discuss the available data on the topic.

## The ripple effects of the covid-19 pandemic on nutrition and lifestyle habits of children

Studies reporting the indirect effects of the COVID-19 crisis on nutrition and lifestyle in children are still scarce and were mainly carried out in high- or middle-income countries. An Italian survey conducted during 3 weeks of home confinement included 41 obese children aged 6–18 years [7]. It showed that intakes of potato chip, red meat and sugary drinks increased significantly during the lockdown (*P*-value range: 0.005 to < 0.001), while time spent in sports activities decreased by 2.3 (±4.6 SD) hours/week (*P* = 0.003) and screen time increased by 4.8 (±2.4 SD) hours/day (*P* < 0.001). Similarly, an international survey conducted in Italy, Spain, Chile, Colombia and Brazil among 820 adolescents reported a significant increase in the consumption of fried and sweet foods during COVID-19 restrictions with greater adherence to unhealthy food among adolescents whose mothers had low education [8]. Another Italian survey conducted during confinement included 3533 respondents aged between 12 and 86 years [9]. The population group aged 12–17 years reported an increase in junk food consumption and a lower adherence to the Mediterranean diet when compared to the group aged 18–30. In the United States, 1048 families enrolled in a school-based nutrition program responded to an electronic survey covering 4 geographic areas [10]. Overall, 93.5% of respondents reported being food insecure in April 2020 compared to 71.5% in fall 2019, and 41.4% reported a decrease in fruit and vegetable intake because of COVID-19 [10].

On the other hand, an online survey conducted in Poland in May 2020 among 2448 adolescents aged 15–20 years suggested that the pandemic may have brought positive changes promoting the uptake of a better diet [11]. It revealed that health and weight control were the main determinants of food choices of adolescents during the COVID-19 pandemic compared to before the pandemic, outstripping the sensory appeal of food and mood (*p* < 0.0001). One of the largest studies conducted on this topic comes from the NutriNet-Sante cohort which surveyed a population of 37.252 French adults and households between March and May 2020 [12]. The findings suggest that the lockdown led, in a substantial part of the population, to unhealthy nutritional and lifestyle behaviors: decreased physical activity (53%), increased sedentary time (63%), increased snacking (21%), decreased consumption of fresh foods (27%), increased consumption of sweets (22%), eating in response to boredom (18%) or anxiety (10%) with a weight gain of 1.8 kg on average for 35% of the respondents. However, the lockdown also created an opportunity for some people to improve their nutritional behaviors including increased home-made cooking (40%) and increased physical activity (19%). Interestingly, persons with less favorable nutritional trends were more likely to have lower incomes, to be overweight or obese, to have anxiety or depressive symptoms and to have children under 18 years at home [12].

At our University Hospital in Lausanne Switzerland, we conducted a survey in the emergency department in August 2020. Parents of stable patients received a questionnaire on eating and lifestyle habits during but also 3 months after the semi-confinement carried out in Switzerland between mid-March and end of April 2020. The questionnaire was filled out by 128 families, including a non-negligible proportion of migrants. The sample was equally distributed between genders and one third of children was younger than 4 years. *During confinement*, 40% of children reported eating and snacking more than usual. Time spent on screens increased for 75% of children and up to 100% for adolescents. More alarming was the percentage of children under the age of 4 watching screens: 73% of boys and 41% of girls. As for physical activity, the proportion of children spending less than 2 h of light physical activity per day doubled during confinement. One third of parents estimate that their child gained excess weight during this period, but their previous growth parameters were not available for an objective comparison. The survey also revealed that a significant proportion of children (58%) suffered changes in their behavior: sleep disturbances, stress and anxiety. The most concerning finding is the persistence of the majority of these bad habits more than 3 months *after the lockdown*, for 20% of children regarding snacking and for 37% of them regarding screen time.

## Potential consequences on children and required actions

Even though the impact of the COVID-19 pandemic on children has yet to be fully measured, the first available data and estimates from international organizations give us some clues as to how this crisis could affect the nutrition and lifestyle of children. Overall, there are concerns about a risk of increased pediatric obesity mainly in middle- and high-income countries with risk of a shift towards an “obesity pandemic” while undernutrition is expected to deepen in poor countries threatening to put years of global progress to end preventable child deaths in serious jeopardy [4]. In fact, in low-income countries where underweight and overweight coexist, undernutrition is expected to increase by an additional 6.7 million children in 2020 especially in regions already affected by humanitarian crisis, adding to the 47 million children under 5 already suffering from wasting, and the 144 million children affected by stunting mostly in Asia and Africa [4]. More than 10 000 additional child deaths per month are expected during this period [3]. Social and income inequalities represent a major risk factor for poor outcomes in poor and rich countries, a fact that has been terribly highlighted by the current health crisis.

The poor diet quality and sedentary lifestyle potentially acquired during the pandemic may not be easily reversible for children and their parents. Infancy and early childhood are a key period for learning healthy eating habits that accompany us throughout our lives and into adulthood. Furthermore, inadequate nutrition at an early age could have life-long repercussions. Nutrition is intrinsically linked to the immune system and to disease susceptibility. Poor diets cause huge costs in health and human lives [5]; while wasted children are at increased risk of death from infectious diseases [4], non-communicable diseases linked to obesity place a heavy burden on public health [8].

Although all children are casualties of the COVID-19 pandemic, vulnerable groups are at higher risk including migrants, refugees, children with mental health issues as well as households with low income or low education level. Migrants often face a dual burden of malnutrition with increased risk of undernutrition upon arrival in the host country and increased risk of overweight/obesity once settled in a westernized environment [13]. In late 2015, we performed a prospective study among a sample of 29 migrant children followed in our migrant clinic at Lausanne University Hospital to evaluate their eating habits and adaptation process [13]. It revealed that unhealthy snacking and consumption of sweet beverages tripled after immigration while intake of vegetables and fruits declined. Most children came from Middle East (Syria, Irak), Kosovo and Africa. The COVID-19 crisis could exacerbate these nutritional trends due to the economic burden.

Further studies are required to measure the impact of this pandemic on the nutritional habits and status of children in different contexts. They should assess whether unhealthy nutritional habits are maintained in the long term or even improved, and also objectively assess children’s nutritional parameters and compare them to the pre-pandemic era. Available studies do not provide information on changes in body mass index or other growth parameters to our knowledge.

Several international and pediatric organizations have issued a call to action to mitigate the effects and inequities that are widening in the shadow of COVID-19 and to preserve global child right to optimal health and development. Response plans should incorporate social-protection programs, public awareness campaigns and nutrition education programs focusing on precarious groups. Pediatricians and child health professionals are key players in terms of prevention and education of families. Financial assistance to poor families is crucial given the low affordability of healthy diets who remain out of reach for numbers of families. The battle against inequalities and the various forms of malnutrition is a public health issue common to all countries.

## Conclusion

The fallout from the COVID-19 pandemic goes far beyond that of a viral infection and threatens to undo decades of hard-won progress in pediatrics. While it is important to understand the direct effects of the virus which affects a small proportion of children, we should not forget about all the collateral damages that this pandemic could have on many children. The impact on nutrition and lifestyle is one of the submerged parts of this iceberg with potential intergenerational consequences. Nutrition and lifestyle should be a core component of a response plan to such a pandemic particularly for marginalized groups. The true burden of the pandemic in children is yet to be unveiled.

## Data Availability

The datasets used and/or analysed during the current study are available from the corresponding author on reasonable request.
